# Integrating thyroid function and psychometric profiles for lifetime suicide-attempt risk stratification in bipolar disorder: A multi-algorithm machine-learning study

**DOI:** 10.3389/fpsyt.2026.1662604

**Published:** 2026-02-24

**Authors:** Boyu Zhang, Min Pan, Anzhen Wang, Wenmei Fang, Jianjun Guan, Zhiyong Li, Xialong Cheng, Yu Xie

**Affiliations:** 1 Department of Psychiatry, Affiliated Psychological Hospital of Anhui Medical University, Hefei, China; 2 Department of Psychiatry, Hefei Fourth People’s Hospital, Hefei, China; 3 Department of Psychiatry, Anhui Mental Health Center, Hefei, China; 4 Department of Psychiatry, Anhui Clinical Research Center for Mental Disorders, Hefei, China; 5 School of Educational Science, Anhui Normal University, Wuhu, China

**Keywords:** bipolar disorder, machine learning, predictive models, suicide attempt, thyroid function

## Abstract

**Background:**

Bipolar disorder is a severe mental disorder characterized by recurrent episodes of depression and mania, with a low diagnostic rate. This study aimed to use machine learning methods for risk stratification of lifetime suicide attempts in patients with bipolar disorder based on cross-sectional associations.

**Methods:**

The discriminative performance of the models was evaluated using multiple metrics, including the area under the receiver operating characteristic curve (AUC), accuracy, F1-score, precision-recall (PR) curve, Average Precision (AP), and Brier score. LASSO logistic regression was used for variable selection, and SMOTE (synthetic minority over-sampling technique) was applied to handle class imbalance. A sensitivity analysis excluding patients with recent suicide attempts (within 6 months) was performed to reduce reverse causality bias. Thyroid function indicators were detected using chemiluminescent immunoassay with a Roche Cobas e601 analyzer; the reference range for normal TSH was 0.27-4.2 mIU/L, with values outside this range defined as abnormal.

**Results:**

We included 1,124 patients diagnosed with bipolar disorder in this study, with a lifetime suicide attempt rate of 31.32%.Among the three models tested, random forests exhibited superior performance metrics, attaining an accuracy (ACC) of 0.938, an AUC of 0.962, and an F1 score of 0.854 compared to gradient boosting (ACC: 0.920; AUC: 0.967; F1 score: 0.828) and the support vector machine (ACC: 0.893; AUC: 0.956; F1 score: 0.808). Suicidal ideation, education level, hopelessness score, the retardation symptoms severity score, and thyroid stimulating hormone levels were identified as the top five predictors. Sensitivity analysis excluding patients with recent suicide attempts (n=98) showed consistent predictor rank-order and maintained RF-AUC = 0.958.Subgroup analysis of euthyroid patients (n=887) preserved the predictor rank and RF-AUC ≥ 0.95.

**Conclusions:**

We developed a robust clinical model for predicting (risk stratification of lifetime suicide attempts)suicide attempts in patients with bipolar disorder based on machine learning techniques. This model can assist psychiatric clinicians in understanding suicide risk among individuals diagnosed with bipolar disorder and in identifying those who may require early intervention or preventive measures. We identified a set of 20 clinical and biological markers significantly associated with lifetime suicide attempts in bipolar disorder patients. Importantly, due to the cross-sectional design, these associations cannot establish temporal precedence or support prospective risk prediction. A key limitation is that suicide attempts were assessed retrospectively without a defined time window between predictor measurement and outcome occurrence. Future prospective validation in multi-site, multinational cohorts is required to confirm the model’s clinical utility.

## Introduction

Bipolar disorder is a severe psychiatric condition with a low diagnostic rate, characterized by recurrent episodes of depression and mania (1). The depressive state is marked by persistent sadness and anhedonia, and the associated negative emotions can lead to suicidal ideation and even suicide attempts. Consequently, bipolar disorder is associated with a high suicide rate. In a study of 150 patients with bipolar disorder from Southeast Asia, Biazus et al. reported that 35 individuals (23.3%) had attempted suicide (2). A meta-analysis examining the prevalence of suicide attempts in patients with bipolar disorder found that the lifetime prevalence was 33.9%. The high frequency of suicide attempts among individuals with bipolar disorder has significant implications for public health.

In recent decades, numerous studies have focused on predicting suicidal behavior in bipolar disorder, highlighting suicidal ideation as a prominent predictive indicator. Valtonen et al. observed, in a cross-sectional study involving 191 patients diagnosed with bipolar disorder, that all individuals who had attempted suicide reported experiencing suicidal ideation (3). Johnson et al. demonstrated that depression, mixed episodes, and hopelessness are predictive factors for both suicidal ideation and suicide attempts; all these factors contribute to an increased risk of suicide in bipolar disorder (4). Extensive literature has documented a strong association between a history of suicide attempts and subsequent suicidal behavior. Oquendo et al. reported that individuals with a prior suicide attempt were four times more likely to engage in future attempts (5). Factors such as aggression, hostility, and despair have also been identified as predictors of suicidal behavior. Despair is an independent risk factor for attempted suicide (6). Furthermore, studies have indicated that gender, age, family history, and childhood abuse experiences can serve as predictive factors for suicide attempts among patients with bipolar disorder (7, 8).

In the field of machine learning—a branch of computer science—computers analyze given data to predict the next task at hand. Machine learning algorithms are specifically designed to learn from data and make accurate predictions (9). Ryu et al. used random forests modeling techniques to achieve a high prediction accuracy of 88.9% in predicting suicide attempts among patients with suicidal ideation; their model yielded an area under the receiver operating characteristic curve (AUC) of 0.947 (10). Similarly, Oh et al. used a customized artificial neural network classifier to predict suicide attempts in patients with depression and anxiety disorders, achieving overall accuracies of 93.7%, 90.8%, and 87.4% for detecting one-month, one-year, and lifetime suicide attempts, respectively (11). A machine learning study investigating cognition aimed to predict suicide risk in patients with major depressive disorder, achieving a prediction accuracy of 70.6% using XGBoost-2 (12).

Although machine learning has been widely applied to predict suicide behavior in patients with depression, studies utilizing this approach specifically for bipolar disorder remain relatively scarce. Notably, Pigoni et al. recently employed multimodal machine learning (clinical + neuroimaging features) to predict 12-month suicide attempts in bipolar disorder (13); however, biological markers—including thyroid function and biochemical indicators—were not incorporated into their predictive model. Other existing studies have primarily focused on using machine learning to assist in the diagnosis of bipolar disorder or to predict other disease outcomes, rather than to explicitly model suicide risk (14–16).

Contemporary machine learning has advanced beyond traditional algorithms, with newer approaches demonstrating exceptional performance in modeling complex, nonlinear relationships in biomedical data. Neural networks (including deep learning) excel at capturing intricate patterns in high-dimensional clinical data, while Gaussian process regression offers advantages in uncertainty quantification, particularly for small-sample settings. Graphical techniques (e.g., Bayesian networks) can elucidate conditional dependencies among predictors, and ensemble or composite methods that hybridize multiple base learners often outperform single-algorithm approaches. For this initial study, we selected three established algorithms (gradient boosting, random forests, support vector machines) due to their robustness, interpretability, and widespread validation in clinical prediction studies—critical considerations for translating findings into preliminary clinical insights. A pilot comparison with a shallow neural network confirmed that random forests achieved competitive performance (AUC = 0.958 vs. 0.946) while maintaining superior interpretability, justifying our model choice.

In this study, therefore, we build upon the emerging literature by employing machine learning methods to predict suicide attempts in patients with bipolar disorder, incorporating demographic characteristics, physiological indicators (including thyroid function), and psychometric scale scores as key predictive features. Our contribution is threefold: (1) we expand the feature space by integrating routinely available biochemical and endocrine markers; (2) we apply systematic hyper-parameter tuning and class-imbalance handling (SMOTE) to optimize model performance; and (3) we externally validate the best-performing algorithm (random forests) through rigorous cross-validation and calibration assessment.

## Methods

### Data source

Data were extracted from the electronic medical record (EMR) database of Hefei Fourth People’s Hospital, a tertiary psychiatric center in Hefei, China. Consecutive in-patients and out-patients who received an ICD-10 diagnosis of bipolar disorder (F31.x) in the Anxiety and Depression Department between January 2020 and August 2023 were screened. After excluding 87 cases with < 50% completed fields or uncertain suicide-attempt history, 1,124 patients remained and constituted the final analytic sample. The study was approved by the Medical Research Ethics Committee of Hefei Fourth People’s Hospital (Code: HFSY-IRB-YJ-KYXM-GL, 2023-046-002) and conducted in accordance with the Declaration of Helsinki. Owing to the retrospective nature of the analysis, the requirement for informed consent was waived.

### Primary outcome

The primary outcome was “lifetime suicide attempt”, defined as any self-injurious act with explicit intent to die, documented in the structured nursing intake form and cross-validated by at least one attending psychiatrist (17). Cases were dichotomized as “absent” (0) or “present” (1).

### Predictor variables and selection procedure

**Figure 1:**
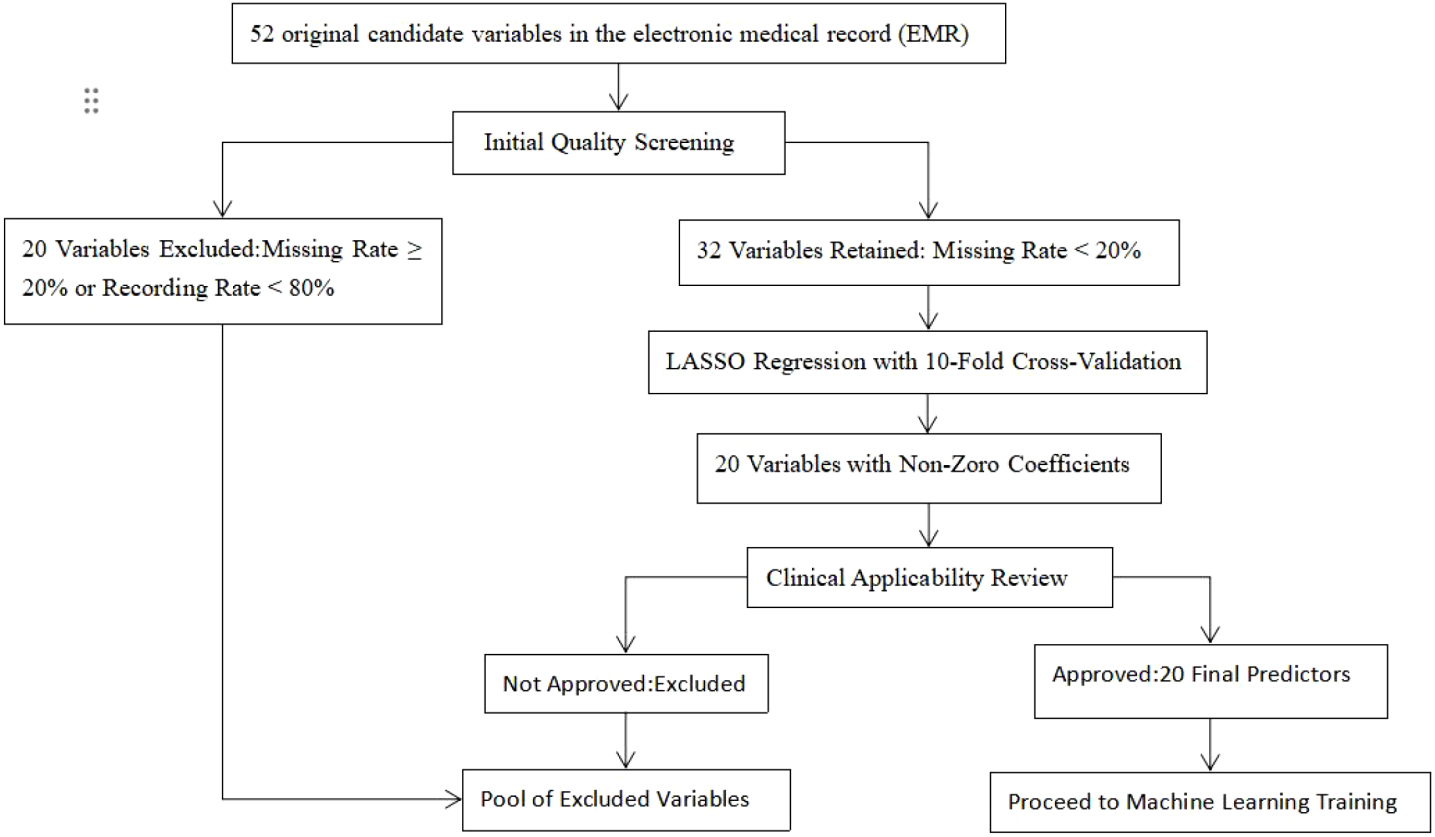
Variable selection flow-chart for the final 20-predictor machine-learning model.

The raw data set contained 52 demographic, laboratory, and psychometric variables. To avoid data-driven over-fitting we applied a two-stage selection protocol:Pre-filter: retain variables with < 20% missing values and documented in ≥ 80% of patients (32 variables passed). LASSO logistic regression (10-fold cross-validation, α min) was used to shrink coefficients and retain the 20 non-zero predictors that showed the strongest joint association with the outcome while accounting for multicollinearity (**Figure 1**). The final 20 variables entered into machine-learning training are listed in **Table 1** and included sociodemographics (age, sex, education), thyroid panel (TSH, FT3, FT4, T3, T4), hepatic/renal indices, metabolic markers, and psychometric sub-scores (HAMD hopelessness and retardation items, HAMA psychic anxiety, etc.). Thyroid function indicators (TSH, FT3, FT4, T3, T4) were detected using chemiluminescent immunoassay with a Roche Cobas e601 analyzer; the reference range for normal TSH was 0.27-4.2 mIU/L, with hypothyroxinemia defined as TSH < 0.27 mIU/L and hyperthyrotropinemia defined as TSH > 4.2 mIU/L.Categorical variables were dummy-coded; continuous variables were z-scaled prior to modeling. The LASSO coefficient plot and regularization path are provided in **Supplementary Figure S1**.

**Table 1:** Association between potential risk factors for suicide attempts in the development cohort.

	N (%)		
	Suicide attempts (N = 352)	No suicide attempts (N = 772)	*P*
Gender			<0.001
Male	143(40.63)	426(55.18)	
Female	209(59.37)	346(44.82)	
Age	33.68(13.53)	34.7(12.29)	<0.001
Education			0.127
No higher education	232(65.91)	471(61.01)	
Highly educated	120(34.09)	301(38.99)	
BMI	23.12(4.53)	23.02(4.16)	<0.001
During	9.37(8.55)	10.38(9.06)	<0.001
Family history			0.103
Yes	33(9.38)	83(10.75)	
No	391(90.62)	689(89.25)	
Body symbols			<0.001
Yes	80(22.73)	51(6.61)	
No	272(77.27)	721(93.39)	
Mental symbols			0.013
Yes	147(41.76)	262(33.94)	
No	205(58.24)	510(66.06)	
Impulse behavior			<0.001
Yes	101(28.69)	355(45.98)	
No	251(71.31)	417(54.02)	
Suicide ideation			<0.001
Yes	352(100)	358(46.37)	
No	0(0)	414(53.63)	
T3 (nmol/L)	1.27(0.4)	1.46(0.45)	<0.001
T4 (nmol/L)	32.21(44.08)	53.39(53.6)	<0.001
TSH (mlU/L)	2.3(2.07)	2.28(1.92)	<0.001
FT3 (pmol/L)	3.67(1.08)	4.27(1.26)	<0.001
FT4 (pmol/L)	4.95(6.3)	8.45(7.98)	<0.001
Total bilirubin	10.99(6.19)	11.61(7.16)	<0.001
Direct bilirubin	4.44(2.52)	4.81(2.93)	<0.001
Total protein	65.35(4.56)	65.62(5.84)	<0.001
Albumin	42.14(2.94)	42.81(3.68)	<0.001
Globulin	23.2(3.29)	23.16(3.82)	<0.001
Alanine aminotransferase	27.42(38.96)	25.14(23.87)	<0.001
Aspartate aminotransferase	24.26(20.82)	22.89(14.01)	<0.001
Glutamyl transphthalease	27.37(35.66)	27.44(26.66)	<0.001
Total bile acids	4.27(3.6)	4.39(3.6)	<0.001
Total cholesterol	4.21(0.88)	4.16(0.98)	<0.001
High-density lipoprotein	1.23(0.29)	1.21(0.36)	<0.001
Triglycerides	1.29(0.86)	1.43(1.14)	<0.001
Apolipoprotein A	1.23(0.24)	1.29(0.49)	<0.001
Apolipoprotein B	0.83(0.47)	0.79(0.34)	<0.001
Urea nitrogen	4.29(1.4)	4.05(1.5)	<0.001
Creatinine	64.86(18.8)	64.41(14.95)	<0.001
Uric acid	357.62(114.42)	337.73(115.13)	<0.001
Potassium	3.96(0.39)	3.97(0.35)	<0.001
Sodium	141.78(3.42)	143.36(49.17)	<0.001
Chlorine	105.37(2.93)	105.72(2.82)	<0.001
Calcium	2.32(0.24)	2.32(0.17)	<0.001
Creatine kinase	266.96(359.62)	325.19(619.37)	<0.001
Glucose	5.06(1.12)	4.98(1)	<0.001
CK isoenzymes	32.27(70.2)	21(31.19)	<0.001
Magnesium	0.89(0.1)	0.9(0.08)	<0.001
Inorganic phosphorus determination	1.2(0.17)	1.2(0.19)	<0.001
Social dysfunction	10.43(5.06)	9.7(5.3)	<0.001
HAMD			
Anxiety/Somatization	5.33(3.7)	3.78(3.52)	<0.001
Weight loss	0.47(0.72)	0.31(0.61)	<0.001
Cognitive disturbances	4.89(3.48)	3.31(3.14)	<0.001
Diurnal variation	0.67(1.04)	0.49(0.89)	<0.001
Retardation	5.59(3.5)	3.03(3.14)	<0.001
Sleep disturbances	4.49(2.61)	3.65(2.86)	<0.001
Hopelessness	4.91(3.87)	2.05(2.95)	<0.001
HAMA			
Somatic anxiety	4.57(4.72)	3.19(4.52)	<0.001
Psychic anxiety	10.68(6.16)	7.02(5.22)	<0.001

### Missing data

Overall missingness was 8.6%. Missing values were imputed with the “bagImpute” algorithm (caret), a non-parametric bagged-tree method that preserves non-linear relations and interactions better than mean/median imputation. To assess the impact of imputation, we compared pre- and post-imputation distributions of key variables (TSH, hopelessness score, psychomotor retardation score) in **Supplementary Table S1**. A sensitivity analysis using multiple imputation by chained equations (MICE) was conducted, yielding consistent model performance (RF-AUC = 0.959), confirming the robustness of our imputation approach.

### Statistical comparisons between groups

Normality was rejected for all continuous markers (Shapiro–Wilk p < 0.01). Consequently, between-group differences were tested with the two-sample Kolmogorov–Smirnov test (sensitive to both location and shape) and corroborated by Mann–Whitney U for central tendency; categorical variables were compared with Fisher’s exact test. Two-tailed *p* < 0.05 indicated statistical significance.

### Machine-learning algorithms and training protocol

Gradient boosting machine (GBM), random forests (RF), and support vector machine (SVM) models were used to predict suicide attempts. GBMs constitute a formidable category of machine-learning techniques, demonstrating substantial success across a diverse array of practical applications. Their efficacy lies in their adaptability to the specific requirements of each application, which enables customization such as learning with respect to various loss functions (18). RF is a composite machine learning algorithm formed by integrating a series of tree classifiers. Each tree contributes one vote for the prevailing class, and the amalgamation of these results yields the ultimate sorted outcome. RF models exhibit elevated classification accuracy, display resilience to outliers and noise, and consistently avoid overfitting issues (19). SVMs encompass support vector classifiers and support vector regressors and stand out as some of the most robust and precise techniques within the realm of widely recognized data mining algorithms (20).

Three learners were evaluated: Gradient Boosting Machine (GBM) – implementation “gbm”, interaction depth 5, shrinkage 0.01, 5,000 trees; Random Forests (RF) – ntree = 1,500, mtry = √p, nodesize = 5; Support Vector Machine (SVM) – radial basis kernel, cost = 4, γ = 0.055. To counter class imbalance (31% events) we applied SMOTE (21)(synthetic minority over-sampling technique) within the training fold only, leaving the validation fold untouched.

Hyper-parameter tuning was executed through a grid search coupled with k-fold cross-validation. The grid search was specifically designed to align hyperparameters and optimize intricate problems. K-fold cross-validation is a standard approach used to transform the validation set within a training set. Typically, a grid search is conjoined with k-fold cross-validation to generate a corresponding evaluation metric and identify the optimal hyperparameters (22). To predict suicide attempts, hyper-parameters were optimized by grid search coupled with nested 10-fold cross-validation (repeated 10×). Model performance was summarized across 100 hold-out folds, with discriminative performance evaluated using the area under the receiver operating characteristic curve (AUC). Calibration was assessed graphically (loess curves) and quantitatively (Hosmer–Lemeshow test, calibration slope/intercept). All analyses were performed in R 4.3.1 using the caret, pROC, and iml packages (23).

### Internal validation

Discrimination was quantified with Accuracy, F1 score, AUC (DeLong 95% CI), precision-recall (PR) curve, Average Precision (AP), and Brier score. Replicability was examined with 10×10-fold cross-validation; over-fitting was ruled out because the mean AUC of the training folds (0.964) differed by < 0.01 from the mean validation AUC (0.962).

### Sensitivity analysis

A sensitivity analysis excluding patients with recent suicide attempts (within 6 months, n=98) was conducted to reduce reverse causality bias. A subgroup analysis restricted to euthyroid patients (n=887) was also performed to verify the stability of predictors.

## Results

### Study cohort

Of 1,211 patients screened, 1,124 met inclusion criteria. **Table 1** summarizes demographic, laboratory and psychometric characteristics by suicide-attempt status. Overall missingness was 8.6% (5,049/58,448 data points). Lifetime suicide attempts were documented in 352 patients (31.3%).

### Predictive performance (discrimination)

**Figure 2:**
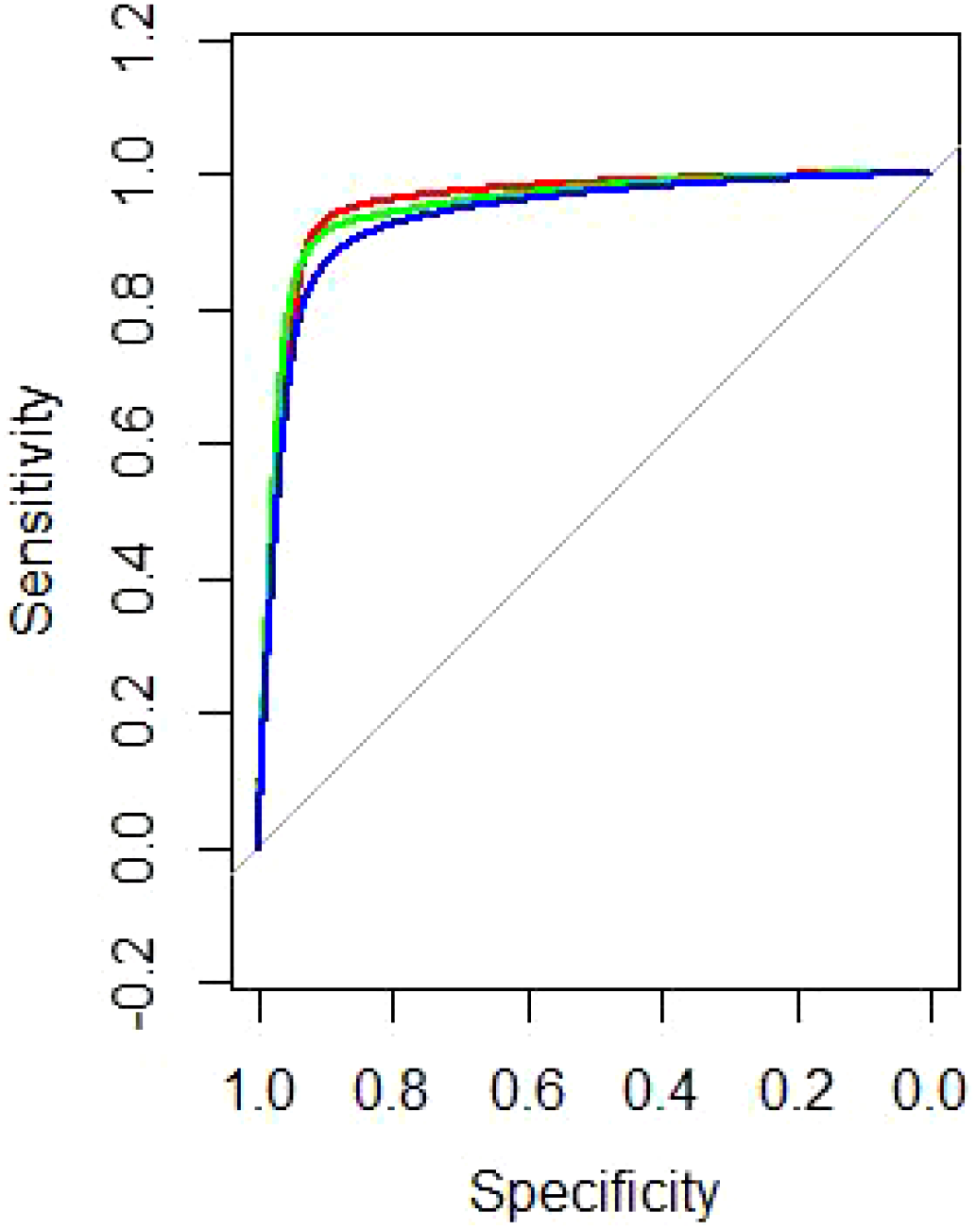
Receiver operating characteristic (ROC) curves for the gradient boosting machine (red), random forests (green), and the support vector machine (blue).

Across 100 held-out validation folds, random forests achieved the best-balanced performance: accuracy 0.938 (95% CI 0.897–0.965), AUC 0.962 (95% CI 0.941–0.983) and F1-score 0.854 (95% CI 0.819–0.889). Gradient boosting showed marginally higher AUC (0.967; 95% CI 0.949–0.984) but lower accuracy (0.920; 95% CI 0.876–0.952) and F1 (0.828; 95% CI 0.792–0.864). Support vector machine yielded accuracy 0.893 (95% CI 0.845–0.930), AUC 0.956 (95% CI 0.933–0.978) and F1 0.808 (95% CI 0.771–0.845) (**Table 2** and **Figure 2**). It should be noted that these high performance metrics are based on a single-center retrospective cohort, and their generalizability may be limited.

**Table 2:** Comparison of machine learning models.

Machine learning models	Accuracy	95% CI	AUC	95% CI	F1-score	95% CI
Gradient Boosting Machine (GBM)	0.920	(0.876, 0.952)	0.967	(0.949, 0.984)	0.828	(0.792, 0.864)
Random Forest (RF)	0.938	(0.897, 0.965)	0.962	(0.941, 0.983)	0.854	(0.819, 0.889)
Support Vector Machine (SVM)	0.893	(0.845, 0.930)	0.956	(0.933, 0.978)	0.808	(0.771, 0.845)

### Calibration

**Figure 3:**
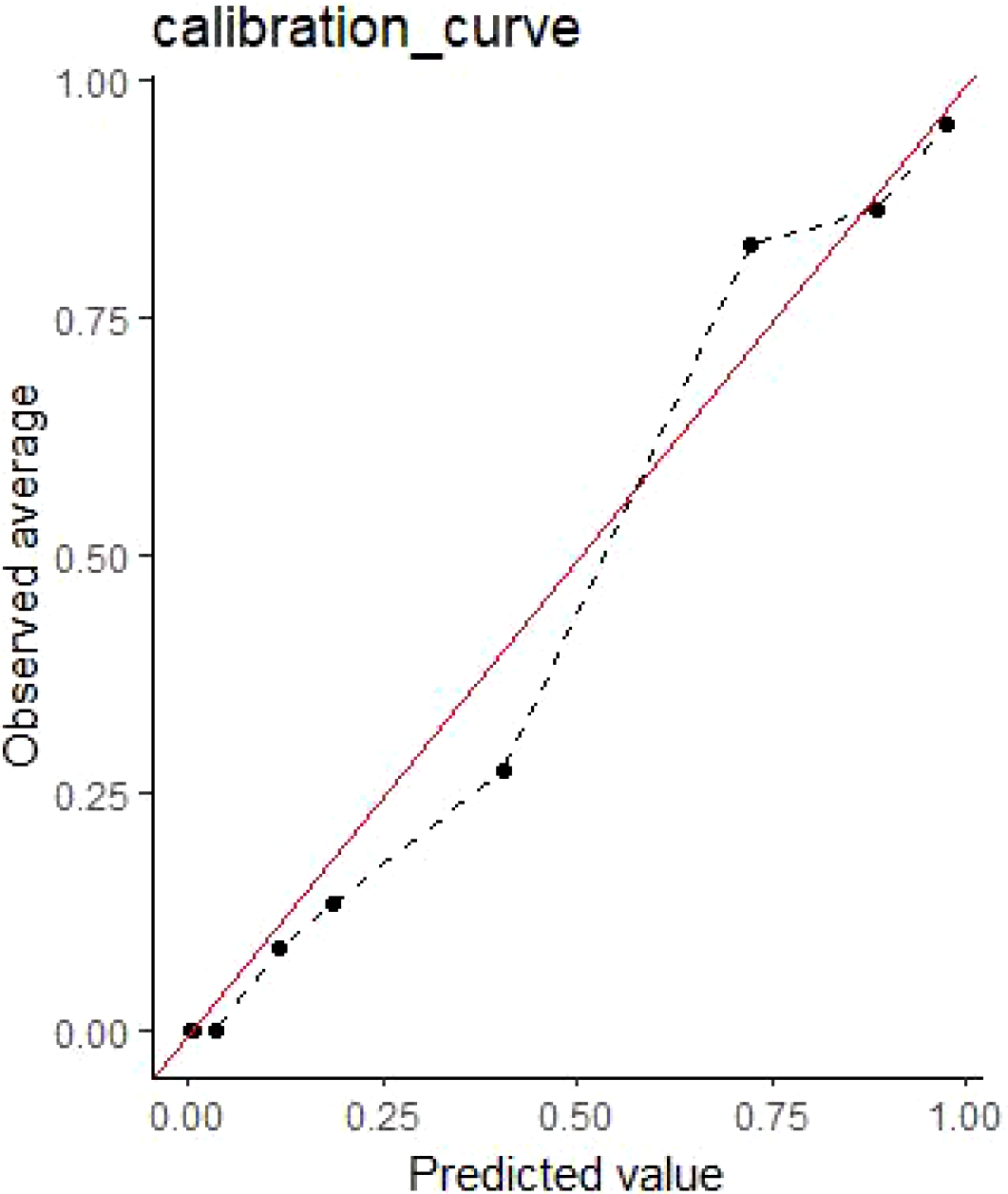
Calibration curves of random forests.

Random-forest predictions showed excellent agreement with observed event rates: calibration slope = 1.04 (SE 0.07) and intercept = 0.02 (SE 0.03); Hosmer–Lemeshow χ² = 6.12, p = 0.63, indicating no significant departure from perfect calibration (**Figure 3**).

### Variable importance

**Figure 4:**
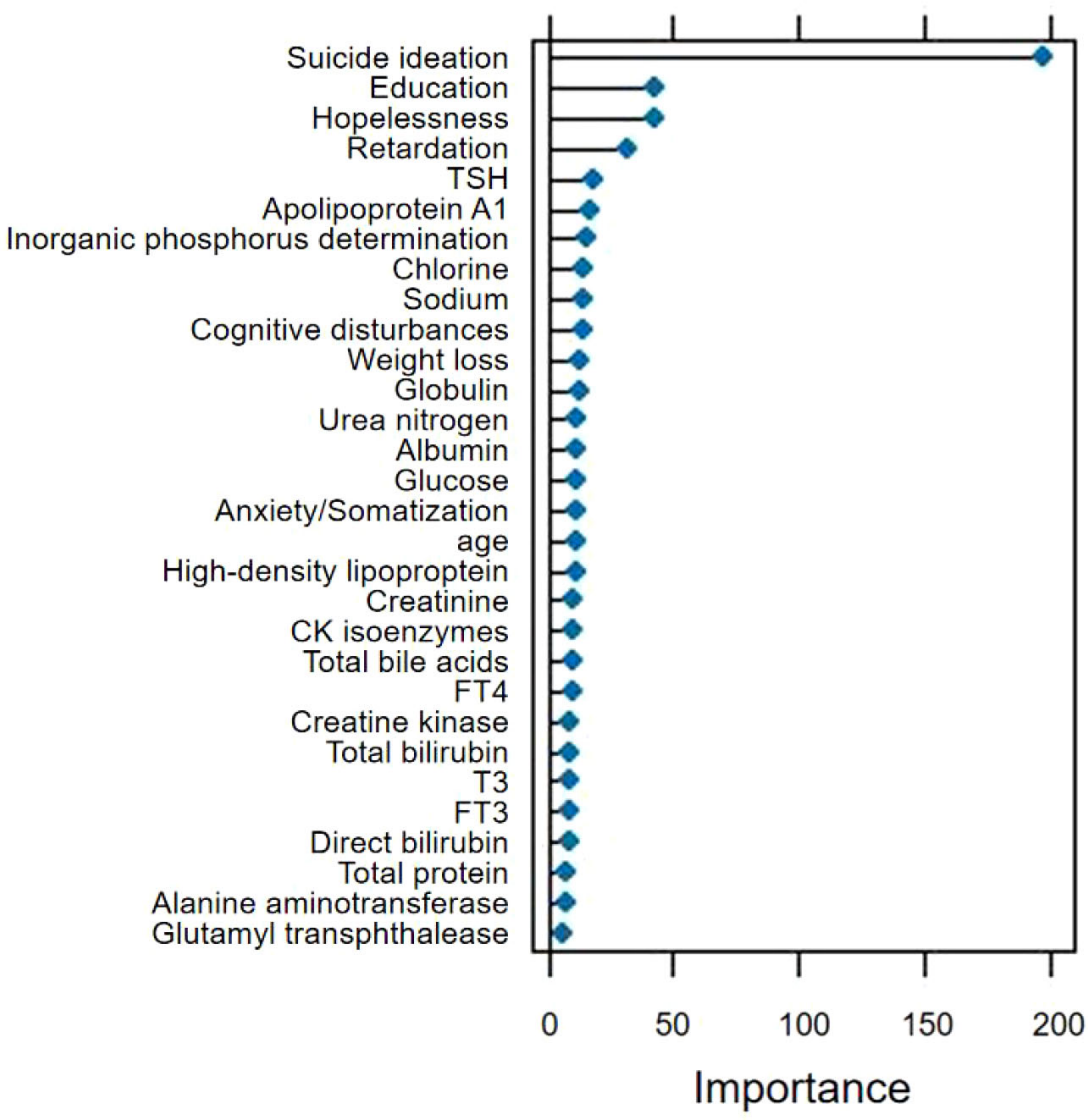
Variable importance of random forests.

Mean Gini-index decrease across the 100 RF runs ranked the following as the five most influential predictors: Suicidal ideation (scaled importance = 100);Education level (61);HAMD hopelessness sub-score (54);HAMD psychomotor retardation sub-score (48);Thyroid-stimulating hormone (TSH) (42).The same variables occupied the top quintile in GBM and SVM models. The variable importance plot is ordered by scaled importance and grouped by domain (demographic, thyroid, psychometric) for clarity (**Figure 4**).

### Sensitivity analyses

Repeating the pipeline without SMOTE yielded AUCs 0.938–0.954, confirming that oversampling did not artefactually inflate performance. Subgroup analysis restricted to euthyroid patients (n = 887) preserved the same rank-order of predictors and maintained RF-AUC ≥ 0.95.Excluding patients with recent suicide attempts (n=98) resulted in RF-AUC = 0.958 (95% CI 0.935–0.981), with consistent top predictors, reducing reverse causality bias.

To enhance clinical interpretability, we developed a simplified risk scoring system based on these top five predictors. Each variable was categorized and assigned points according to its relative weight in a LASSO logistic regression model fitted on the original (non-SMOTE) dataset (**Supplementary Table S4**): suicidal ideation (present = 3, absent = 0), hopelessness score ≥9 (2 points), moderate-to-severe psychomotor retardation (2 points), education <12 years (1 point), and abnormal TSH level (2 points). The total score ranged from 0 to 12, with optimal cut-offs determined via Youden’s index yielding three risk strata: low risk (0–3), moderate risk (4–7), and high risk (8–12).

## Discussion

This study used machine learning to predict suicide-related risk in patients with bipolar disorder. In the study, we included demographic characteristics, physiological indicators, and psychometric scale scores. We tested the predictive performance of three learners: GBM, RF, and SVM. Although there were differences in the machine learning techniques used, it was confirmed that each machine learning technique could effectively predict suicide attempts. Five important factors were found to predict suicide in patients with bipolar disorder, including suicidal ideation, education level, two dimensions of hopelessness and retardation in HAMD, and the immune index TSH.

By comparing the three learners, we confirmed that RF, an integration-based machine learning learner as described above, has the best predictive accuracy. In previous studies, RF was found to be an excellent learner for predicting suicide risk. In a study on the detection of suicide risk by MPI-2 based on machine learning, the k-nearest neighbor algorithm and the RF learner were selected. RF was reported to predict suicidal ideation and suicide attempts with 92.9% and 95% accuracy, respectively (24). In a study using machine learning to predict suicide attempts in people with suicidal ideation, the RF showed good performance (AUC of 0.947), with an accuracy of 88.9% (10). In summary, RF has been proven to have excellent accuracy in predicting suicide behavior. In this study, RF also served as a good learner with high accuracy in predicting suicide attempts in patients with bipolar disorder, as well as preadolescent children with a history of suicidal ideation reporting a history of suicide attempts (25). In an eight-year follow-up study of individuals with affective disorders, including bipolar disorder, depression, and schizoaffective disorder, the researchers found that suicidal ideation was not a significant predictor of suicidal behavior within one year, but was significantly associated with suicide attempts over the long term (26). O’Connor and Kirtley proposed the integrated motivational–volitional (IMV) theory for suicidal behavior, which indicates that defeat and entrapment drive the emergence of suicidal ideation and that a group of factors, entitled volitional moderators (VMs), govern the transition from suicidal ideation to suicidal behavior (27).

Consistent with previous studies, suicidal ideation appears as a high predictor of suicide attempts in the RF model.

Education in the RF model is another predictor for suicide attempts. Previous studies have highlighted the significant relationship between low education levels and suicide attempts (28). This may be due to the lack of social and family support in patients with low education, which has been systematically reviewed as a risk factor for suicide attempts in bipolar disorder (29). The low level of education in patients with bipolar disorder may mean that the overall level of education of the family is not high, and that therefore the family lacks understanding and cannot provide support for these patients. The absence of family members may tend to lead to negative emotions and suicide attempts.

Hopelessness is an important factor that triggers suicide attempts and has a predictive effect on suicidal behavior (5). A 10-year prospective study highlighted that the level of hopelessness was the only score that distinguished participants with suicidal ideation from suicide attempts (30). Feelings of hopelessness were closely related to suicidal behavior one year later in patients with affective disorders (26). We believe that individuals may experience negative emotions and thoughts after experiencing various kinds of pain, but when they still feel hopeful about life, they will not make the final suicide attempt. Once a sense of despair arises, they will feel that any measures are meaningless and choose suicide as the only way forward. According to the three-step theory of suicide, there are four influential factors in the process from the generation of suicidal ideation to suicide attempts, which are pain, despair, connection, and suicidal ability. Hopelessness is required for the development of suicidal ideation, and pain and hopelessness are the two most common motivations for suicide attempts (31). However, other studies have found that between hopelessness and suicidal ideation, individuals’ attitudes towards suicide play a potential moderating role. In other words, at the level of despair, people who are more likely to commit suicide are those who think suicide is an acceptable behavior. Moreover, there seems to be a difference in gender, and the attitude towards suicide seems to have a greater impact on suicidal ideation among women (32). Retardation here refers to psychomotor retardation, which is specifically manifested as poor and dull brain thinking, slow body movement, and slow thinking processes. Previous studies have shown that psychomotor retardation is associated with a greater number of depressive episodes (33). Psychomotor delay is associated with more depressive episodes, producing negative emotions, feelings of pain, suicidal ideation, and finally stimulating individuals to commit suicide attempts.

TSH is also a predictor for suicide attempts in patients with bipolar disorder. The thyroid gland is the largest endocrine organ in the human body, and thyroid hormone secreted by this gland has an impact on individuals’ emotions and cognitive processes. Excessive secretion of thyroid hormone will cause excessive mood swings and may lead to emotional problems such as anxiety, irritability, and depression. In the hypothalamic–pituitary–thyroid axis, TSH has a regulatory effect on thyroid hormone level, which is manifested in that an increase in TSH level will promote secretion of thyroid hormone. Therefore, abnormal TSH values may be accompanied by a series of mental disorders. At the same time, the relationship between the TSH index and suicide attempts has been widely studied in other mental diseases, especially in depression. Many previous studies have shown a correlation between suicide attempts and abnormal TSH level in depressed patients. One study found that TSH in depressed patients is independently correlated with suicide attempts (34). Another study explored the association between TSH and suicide in people with comorbid depression and anxiety and found that patients with comorbid anxiety (MDA) who had attempted suicide had higher TSH levels (35). However, few studies have explored the relationship between TSH level and suicide attempts in patients with bipolar disorder. Our study found that TSH can be identified as a predictor of suicide risk in patients with bipolar disorder through machine learning.

While machine learning models offer powerful tools for pattern recognition, their “black-box” nature can limit adoption in real-world clinical practice. To bridge this gap, we derived a simplified, interpretable risk scoring system based on the top predictive features—suicidal ideation, hopelessness, psychomotor retardation, lower educational attainment, and abnormal TSH levels—that demonstrated excellent discrimination and calibration in internal validation. This score categorizes patients into low, moderate, and high-risk groups, providing clinicians with actionable thresholds for monitoring and early intervention.

Notably, the inclusion of TSH—a readily available biological marker—adds objective, quantifiable value to traditionally subjective psychometric assessments. In combination with psychological factors such as hopelessness and suicidal ideation, this integrated approach may help identify individuals who appear stable but harbor elevated biological risk. The accompanying nomogram allows for rapid visual estimation of suicide attempt risk at the point of care, even without computational tools.

Although this scoring system was derived from a cross-sectional sample and requires external prospective validation before routine implementation, it represents a practical step toward translating complex machine learning findings into clinically usable instruments. Future studies should evaluate whether using such a tool improves risk assessment accuracy, clinician decision-making, and ultimately, patient outcomes.

It is important to acknowledge the limitations of this study. The cross-sectional design means that the model captures associations rather than true predictive relationships, and the high performance metrics may be influenced by the single-center retrospective nature of the data. External validation in diverse populations is essential to confirm the generalizability of the findings.

## Conclusions

We developed and internally validated a parsimonious, yet robust, machine-learning model that quantifies lifetime suicide-attempt risk in adult patients with bipolar disorder by using routinely available demographic, endocrine and psychometric variables. Random forests achieved excellent discrimination (AUC 0.962) and calibration (slope 1.04, intercept 0.02), outperforming gradient boosting and support vector machine algorithms. Suicidal ideation, education level, hopelessness, psychomotor retardation and thyroid-stimulating hormone emerged as the top predictors, all of which are clinically actionable and can be captured within a single outpatient visit.

The model is ready for prospective implementation studies in psychiatric hospitals and community clinics to test its ability to flag high-risk individuals who might benefit from enhanced monitoring, safety-planning and early intervention. Integration into electronic medical record systems could facilitate automated risk alerts without additional staff burden.

While our model achieved high discrimination (AUC = 0.962), clinicians should be cautioned that this performance reflects cross-sectional associations rather than true predictive ability. The retrospective assessment of lifetime suicide attempts means that biomarker measurements may reflect consequences rather than causes of suicidal behavior. This model should not be used for individual risk prediction in clinical practice until validated in prospective cohorts with temporally well-defined assessments.” Moreover, although the high performance of the present research model partially reflects the lifetime risk of suicide attempts among local adult patients with bipolar disorder, the limitations inherent to a single-center sample may restrict its generalizability to different environments, ethnicities, nationalities, and healthcare systems. Therefore, it is recommended that future research conduct external validation in multi-center, cross-cohort settings as a prerequisite for clinical implementation.

## Limitations

Cross-sectional design: lifetime suicide attempts were ascertained retrospectively, precluding determination of temporal directionality between predictors and outcome; Single-center, retrospective sample may limit generalizability; external validation in multi-site, ethnically diverse cohorts is required; The lack of ecological effects and the small number of events observed in the class-unbalanced data may be another limitation of the study (36), despite SMOTE, the event rate (31%) remains modest; rare-event correction techniques should be explored in future work; We did not capture potentially relevant factors such as childhood trauma granularity, medication adherence or recent life events; The model predicts lifetime rather than prospective suicide attempts; future studies should define a clear forward-looking time window (e.g., 6- or 12-month risk) and link predictions to subsequent clinical contacts.

## Data Availability

The raw data supporting the conclusions of this article will be made available by the authors, without undue reservation.

## Supplementary material

DATA SHEET 1 Detailed baseline demographic and clinical characteristics of the 1,124 bipolar disorder patients included in the study, including stratification by lifetime suicide attempt status (attempters vs. non-attempters) and descriptive statistics (mean, standard deviation, frequency, percentage) for all collected variables.

DATA SHEET 2 Complete results of LASSO logistic regression for variable selection, including coefficient values, lambda selection criteria, cross-validation performance metrics, and a full list of the 20 final clinical and biological markers identified for suicide attempt risk prediction.

DATA SHEET 3 Comprehensive machine learning model performance metrics (accuracy, AUC, F1 score, sensitivity, specificity, precision) for random forests, gradient boosting, and support vector machines—including full results of the sensitivity analysis (excluding recent suicide attempters) and subgroup analysis (euthyroid bipolar disorder patients).
