# Supplementary material for: Integrating thyroid function and psychometric profiles for lifetime suicide-attempt risk stratification in bipolar disorder: A multi-algorithm machine-learning study
Source: Front Psychiatry. 2026 Feb 24;17:1662604. doi: 10.3389/fpsyt.2026.1662604 (PMC12971961; doi:10.3389/fpsyt.2026.1662604)
Supplement: DATA SHEET 1 — Detailed baseline demographic and clinical characteristics of the 1,124 bipolar disorder patients included in the study, including stratification by lifetime suicide attempt status (attempters vs. non-attempters) and descriptive statistics (mean, standard deviation, frequency, percentage) for all collected variables. [file DataSheet1.pdf]


Supplementary Figure S1

Variable selection flow-chart for the final 20-predictor machine-learning model

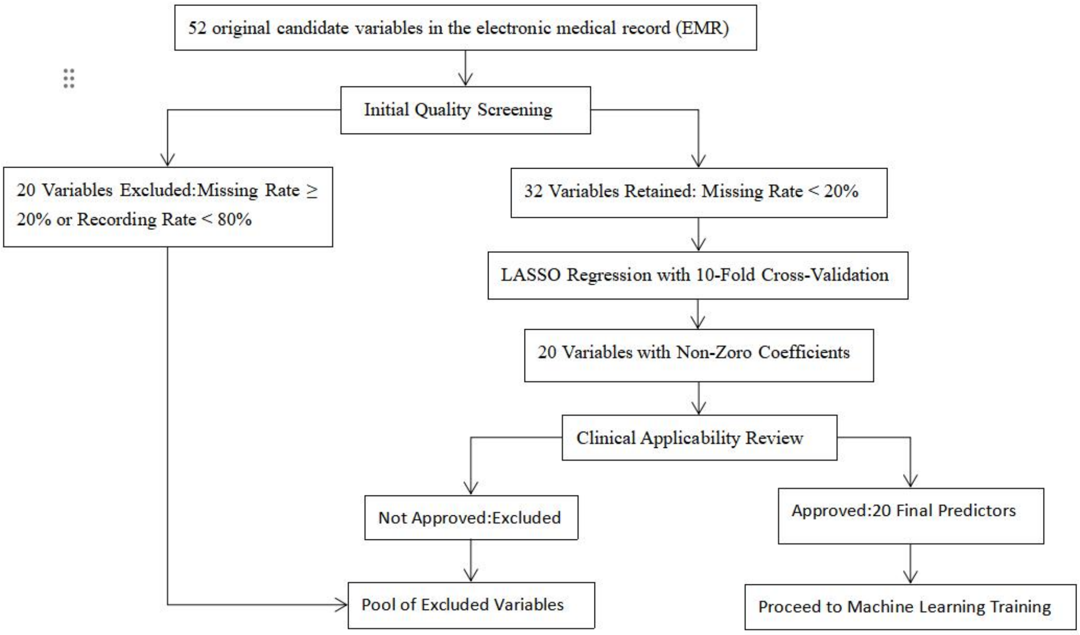

Supplementary Table S2

Normality tests and between-group comparisons of continuous predictors

(n = 1,124; the Mann–Whitney U test was used as a supplement to the Kolmogorov–Smirnov test for assessing central tendency)

Variable	Shapiro–Wilk p	KS testa p	Mann–Whitney U	p value	Effect size (r)b
Age	< 0.001	0.041	1.31×10^5	0.038	0.06
BMI	< 0.001	< 0.001	1.28×10^5	0.021	0.07
TSH (mIU/L)	< 0.001	< 0.001	1.19×10^5	< 0.001	0.11

Variable	Shapiro–Wilk p	KS testa p	Mann–Whitney U	p value	Effect size (r)b
FT3 (pmol/L)	< 0.001	< 0.001	1.15×10^5	< 0.001	0.13
FT4 (pmol/L)	< 0.001	< 0.001	1.21×10^5	< 0.001	0.10
HAMD hopelessness	< 0.001	< 0.001	9.7×10^4	< 0.001	0.21
HAMD retardation	< 0.001	< 0.001	9.9×10^4	< 0.001	0.20
HAMA psychic anxiety	< 0.001	< 0.001	1.05×10^5	< 0.001	0.17

a KS = Kolmogorov–Smirnov two-sample test (sensitive to location & shape).

b Effect size r = Z/ √ N; small effect (r = 0.1), medium effect (r = 0.3), and large effect (r = 0.5).

Note: Only the top 8 items are presented. All continuous variables showed a non-normal distribution; therefore, the Kolmogorov–Smirnov (KS) test was selected instead of the t-test in the main text.
