# Supplementary material for: Integrating thyroid function and psychometric profiles for lifetime suicide-attempt risk stratification in bipolar disorder: A multi-algorithm machine-learning study
Source: Front Psychiatry. 2026 Feb 24;17:1662604. doi: 10.3389/fpsyt.2026.1662604 (PMC12971961; doi:10.3389/fpsyt.2026.1662604)
Supplement: DATA SHEET 2 — Complete results of LASSO logistic regression for variable selection, including coefficient values, lambda selection criteria, cross-validation performance metrics, and a full list of the 20 final clinical and biological markers identified for suicide attempt risk prediction. [file DataSheet2.pdf]

Supplementary Table S4

A Simplified Clinical Risk Scoring System for Lifetime Suicide Attempt in Bipolar Disorder

Variable	Category	Points
Suicidal ideation	Absent	0
	Present (within past month, self-report or clinical assessment)	3
Hopelessness score (Beck Hopelessness Scale)	< 9 (mild/no hopelessness)	0
	≥ 9 (moderate to severe)	2
Psychomotor retardation severity	Mild or none<br>(e.g., CDRS-R item ≤2)	0
	Moderate to severe<br>(e.g., CDRS-R item ≥3)	2
Education level	≥12 years<br>(e.g., high school graduate or higher)	0
	<12 years	1
TSH level	Within normal reference range<br>(typically 0.4–4.0 mIU/L)	0
	Abnormal (either elevated or suppressed)	2

Variable	Category	Points
	(subclinical hypo-/hyperthyroidism)	
	Total Score	0–12

Risk Stratification Thresholds:

Low risk: 0–3 points → Estimated probability: <15%

Moderate risk: 4–7 points → Estimated probability: 15–40%

High risk: 8–12 points → Estimated probability: >40%

Derivation method: Point values were derived by rescaling standardized coefficients from a LASSO logistic regression model fitted on the original (non-SMOTE) dataset. Coefficients were rounded to nearest integer for clinical feasibility. Cut-offs optimized using Youden's index and calibrated against observed event rates.

Clinical use example:
A patient with suicidal ideation (+3), moderate hopelessness (+2), severe psychomotor retardation (+2), education <12 years (+1), and abnormal TSH (+2) has a total score of **10**, placing them in the **high-risk group** (>40% likelihood of lifetime suicide attempt).
